# 3-Methyl-2,6-dinitro-*N*-(3-pent­yl)-4-[(2,3,4-tri-*O*-acetyl-β-d-xylos­yl)amino­methyl]­aniline

**DOI:** 10.1107/S1600536808023192

**Published:** 2008-07-31

**Authors:** Lifei Bai, Xiaoming Wang, Baochang Cai

**Affiliations:** a Jiangsu Key Laboratory of Chinese Medicine Processing, Nanjing University of Chinese Medicine, Nanjing 210029, People’s Republic of China; b State Key Laboratory of Pharmaceutical Biotechnology, School of Life Sciences, Nanjing University, Hankou Road, Nanjing 210093, People’s Republic of China

## Abstract

In the title compound, C_24_H_34_N_4_O_11_, the hexopyranosyl ring adopts a chair conformation. The four substituents are in equatorial positions. The mol­ecule shows an intra­molecular N—H⋯O hydrogen bond.

## Related literature

For related literature, see: Grichar & Dotray (2007 ▶); Kubátová *et al.* (2006 ▶); Wang *et al.* (2008 ▶); Yang *et al.* (2004 ▶).
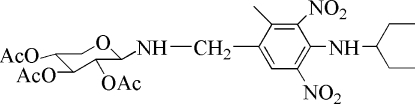


## Experimental

### 

#### Crystal data

C_24_H_34_N_4_O_11_
*M* *_r_* = 554.55
Orthorhombic, 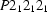

*a* = 7.4100 (15) Å
*b* = 11.044 (2) Å
*c* = 34.106 (7) Å
*V* = 2791.1 (10) Å^3^
*Z* = 4
Mo *K*α radiation
μ = 0.10 mm^−1^
*T* = 293 (2) K
0.30 × 0.20 × 0.10 mm

#### Data collection

Enraf–Nonius CAD-4 diffractometer
Absorption correction: ψ scan (*XCAD4*; Harms & Wocadlo, 1995 ▶) *T*_min_ = 0.969, *T*_max_ = 0.990
2870 measured reflections
2870 independent reflections
1662 reflections with *I* > 2σ(*I*)
3 standard reflections every 200 reflections intensity decay: none

#### Refinement

*R*[*F* ^2^ > 2σ(*F* ^2^)] = 0.067
*wR*(*F* ^2^) = 0.196
*S* = 1.04
2870 reflections
352 parameters
13 restraints
H-atom parameters constrained
Δρ_max_ = 0.17 e Å^−3^
Δρ_min_ = −0.23 e Å^−3^

### 

Data collection: *CAD-4 Software* (Enraf–Nonius, 1989 ▶); cell refinement: *CAD-4 Software*; data reduction: *XCAD4* (Harms & Wocadlo, 1995 ▶); program(s) used to solve structure: *SHELXS97* (Sheldrick, 2008 ▶); program(s) used to refine structure: *SHELXL97* (Sheldrick, 2008 ▶); molecular graphics: *SHELXTL* (Sheldrick, 2008 ▶); software used to prepare material for publication: *SHELXTL*.

## Supplementary Material

Crystal structure: contains datablocks global, I. DOI: 10.1107/S1600536808023192/er2053sup1.cif

Structure factors: contains datablocks I. DOI: 10.1107/S1600536808023192/er2053Isup2.hkl

Additional supplementary materials:  crystallographic information; 3D view; checkCIF report

## Figures and Tables

**Table 1:** Hydrogen-bond geometry (Å, °)

*D*—H⋯*A*	*D*—H	H⋯*A*	*D*⋯*A*	*D*—H⋯*A*
N1—H1*A*⋯O4	0.86	1.92	2.621 (8)	138

**Fig. 1.**
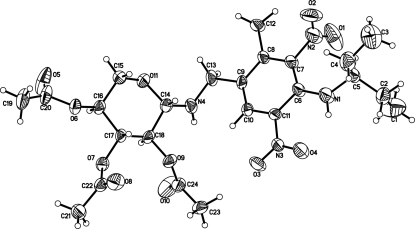
The asymmetric unit of the title compound, showing the atom-labelling scheme. Displacement ellipsoids are drawn at the 50% probability level.

## supplementary crystallographic information

### Comment

Pendimethaline(3,4-dimethyl-2,6-dinitro-*N*-(pentan-3-yl)benzenamine) plays an important role in controlling weeds (Grichar & Dotray, 2007). However, problems such as toxic residues and environmental pollution were protruded increasingly by using amounts of herbicides during the past decades (Kubátová *et al.*, 2006). In the search for a new herbicide with high efficiency and low toxicity, we obtained the title compound. All bond lengths and angles in the title molecule show normal values. The hexopyranosyl ring adopts a chair conformation (Fig. 1). The three acetyl groups are individually planar and occupy equatorial positions (Yang *et al.*, 2004; Wang *et al.*,2008). The torsion angle C14—N4—C13—C9 is 95.8°.

### Experimental

The title compound was prepared from β-*D*-1-amine-2,3,4-tri-O-acetyl-xylosyl with 4-(bromomethyl)-3-methyl-2,6-dinitro-*N*-(pentan-3-yl)benzenamine in dimethylformamide (DMF) in the presence of sodium carbonate. Fine yellow block crystals suitable for single-crystal X-ray diffraction were obtained by slow evaporation of an acetonitrile and ethyl acetate (1:10) solution at room temperature.

### Refinement

In the absence of significant anomalous scattering effects Friedel pairs have been merged.

### Crystal data

**Table:**

C_24_H_34_N_4_O_11_	*F*_000_ = 1176
*M**_r_* = 554.55	*D*_x_ = 1.320 Mg m^−^^3^
Orthorhombic, *P*2_1_2_1_2_1_	Mo *K*α radiation λ = 0.71073 Å
Hall symbol: P 2ac 2ab	Cell parameters from 25 reflections
*a* = 7.4100 (15) Å	θ = 10–13º
*b* = 11.044 (2) Å	µ = 0.11 mm^−^^1^
*c* = 34.106 (7) Å	*T* = 293 (2) K
*V* = 2791.1 (10) Å^3^	Block, yellow
*Z* = 4	0.30 × 0.20 × 0.10 mm

### Data collection

**Table:**

Enraf–Nonius CAD-4 diffractometer	*R*_int_ = 0.0000
Radiation source: fine-focus sealed tube	θ_max_ = 25.1º
Monochromator: graphite	θ_min_ = 1.2º
*T* = 293(2) K	*h* = 0→8
ω/2θ scans	*k* = 0→13
Absorption correction: ψ scan(XCAD4; Harms & Wocadlo, 1995)	*l* = 0→40
*T*_min_ = 0.969, *T*_max_ = 0.990	3 standard reflections
2870 measured reflections	every 200 reflections
2870 independent reflections	intensity decay: none
1662 reflections with *I* > 2σ(*I*)	

### Refinement

**Table:**

Refinement on *F*^2^	Secondary atom site location: difference Fourier map
Least-squares matrix: full	Hydrogen site location: inferred from neighbouring sites
*R*[*F*^2^ > 2σ(*F*^2^)] = 0.067	H-atom parameters constrained
*wR*(*F*^2^) = 0.196	*w* = 1/[σ^2^(*F*_o_^2^) + (0.1035*P*)^2^] where *P* = (*F*_o_^2^ + 2*F*_c_^2^)/3
*S* = 1.04	(Δ/σ)_max_ < 0.001
2870 reflections	Δρ_max_ = 0.18 e Å^−^^3^
352 parameters	Δρ_min_ = −0.23 e Å^−^^3^
13 restraints	Extinction correction: none
Primary atom site location: structure-invariant direct methods	

### Special details

**Table:**

Geometry. All e.s.d.'s (except the e.s.d. in the dihedral angle between two l.s. planes) are estimated using the full covariance matrix. The cell e.s.d.'s are taken into account individually in the estimation of e.s.d.'s in distances, angles and torsion angles; correlations between e.s.d.'s in cell parameters are only used when they are defined by crystal symmetry. An approximate (isotropic) treatment of cell e.s.d.'s is used for estimating e.s.d.'s involving l.s. planes.
Refinement. Refinement of *F*^2^ against ALL reflections. The weighted *R*-factor *wR* and goodness of fit *S* are based on *F*^2^, conventional *R*-factors *R* are based on *F*, with *F* set to zero for negative *F*^2^. The threshold expression of *F*^2^ > σ(*F*^2^) is used only for calculating *R*-factors(gt) *etc*. and is not relevant to the choice of reflections for refinement. *R*-factors based on *F*^2^ are statistically about twice as large as those based on *F*, and *R*- factors based on ALL data will be even larger.

### Fractional atomic coordinates and isotropic or equivalent isotropic displacement parameters (Å^2^)

**Table:**

	*x*	*y*	*z*	*U*_iso_*/*U*_eq_	
N1	0.8178 (10)	0.2626 (5)	0.1700 (2)	0.095 (2)	
H1A	0.9024	0.2234	0.1583	0.114*	
C1	0.8949 (13)	0.0842 (12)	0.2352 (3)	0.142 (5)	
H1B	0.9437	0.0066	0.2422	0.213*	
H1C	0.8294	0.1169	0.2571	0.213*	
H1D	0.9916	0.1381	0.2284	0.213*	
O1	0.6282 (15)	0.4037 (7)	0.2480 (2)	0.166 (5)	
C2	0.7702 (12)	0.0702 (7)	0.2009 (2)	0.080 (2)	
H2A	0.6772	0.0123	0.2079	0.096*	
H2B	0.8382	0.0362	0.1792	0.096*	
N2	0.5848 (15)	0.4284 (7)	0.2134 (4)	0.124 (4)	
O2	0.4388 (11)	0.4255 (7)	0.1997 (4)	0.178 (5)	
N3	1.1129 (8)	0.3679 (5)	0.12461 (15)	0.0628 (15)	
O3	1.2320 (8)	0.4167 (5)	0.10820 (17)	0.0993 (19)	
C3	0.3754 (17)	0.0983 (10)	0.1705 (3)	0.139 (4)	
H3A	0.2938	0.0809	0.1494	0.209*	
H3B	0.3169	0.1501	0.1892	0.209*	
H3C	0.4102	0.0241	0.1831	0.209*	
N4	1.1134 (7)	0.8095 (4)	0.14687 (15)	0.0598 (14)	
H4A	1.2215	0.8042	0.1559	0.072*	
O4	1.0911 (9)	0.2574 (4)	0.12117 (18)	0.104 (2)	
C4	0.5383 (12)	0.1598 (8)	0.1548 (3)	0.100 (3)	
H4B	0.5028	0.2358	0.1428	0.120*	
H4C	0.5921	0.1095	0.1347	0.120*	
O5	1.0104 (14)	1.2551 (5)	0.0281 (2)	0.138 (3)	
C5	0.6794 (10)	0.1850 (6)	0.1869 (2)	0.0673 (19)	
H5A	0.6221	0.2262	0.2090	0.081*	
O6	1.0543 (7)	1.0657 (3)	0.00833 (11)	0.0597 (11)	
C6	0.8401 (10)	0.3854 (5)	0.1690 (2)	0.0645 (19)	
C7	0.7377 (10)	0.4713 (6)	0.1903 (2)	0.071 (2)	
O7	1.3806 (6)	0.9512 (4)	0.02752 (12)	0.0577 (11)	
C8	0.7674 (9)	0.5952 (5)	0.18989 (18)	0.0544 (16)	
O8	1.3595 (8)	0.8031 (5)	−0.01708 (16)	0.0878 (16)	
O9	1.3373 (6)	0.7535 (3)	0.08050 (13)	0.0579 (11)	
C9	0.9162 (9)	0.6406 (5)	0.16961 (17)	0.0497 (15)	
O10	1.5721 (9)	0.8095 (5)	0.1159 (2)	0.128 (3)	
C10	1.0208 (9)	0.5642 (5)	0.14899 (18)	0.0547 (16)	
H10A	1.1179	0.5954	0.1349	0.066*	
O11	0.9818 (6)	0.9650 (4)	0.10992 (12)	0.0625 (12)	
C11	0.9889 (8)	0.4396 (5)	0.14790 (19)	0.0576 (16)	
C12	0.6414 (11)	0.6798 (6)	0.2129 (2)	0.082 (2)	
H12A	0.5486	0.6329	0.2253	0.124*	
H12B	0.5873	0.7369	0.1952	0.124*	
H12C	0.7095	0.7224	0.2324	0.124*	
C13	0.9561 (11)	0.7768 (5)	0.1707 (2)	0.070 (2)	
H13A	0.8515	0.8207	0.1612	0.084*	
H13B	0.9773	0.8013	0.1976	0.084*	
C14	1.0783 (9)	0.8495 (5)	0.1089 (2)	0.0573 (17)	
H14A	1.0031	0.7893	0.0955	0.069*	
C15	0.9289 (9)	1.0028 (6)	0.07198 (19)	0.0647 (18)	
H15A	0.8525	1.0738	0.0739	0.078*	
H15B	0.8606	0.9390	0.0593	0.078*	
C16	1.0952 (9)	1.0320 (5)	0.04803 (17)	0.0544 (16)	
H16A	1.1638	1.0968	0.0608	0.065*	
C17	1.2120 (8)	0.9170 (5)	0.04514 (17)	0.0498 (15)	
H17A	1.1512	0.8556	0.0292	0.060*	
C18	1.2506 (9)	0.8685 (5)	0.08543 (19)	0.0559 (16)	
H18A	1.3310	0.9240	0.0995	0.067*	
C19	1.0118 (14)	1.2105 (7)	−0.0406 (3)	0.105 (3)	
H19A	0.9922	1.2957	−0.0442	0.157*	
H19B	0.9113	1.1662	−0.0511	0.157*	
H19C	1.1202	1.1867	−0.0539	0.157*	
C20	1.0292 (12)	1.1850 (6)	0.0003 (3)	0.080 (2)	
C21	1.6071 (11)	0.9433 (6)	−0.0209 (2)	0.077 (2)	
H21A	1.6419	0.8986	−0.0438	0.115*	
H21B	1.7018	0.9391	−0.0017	0.115*	
H21C	1.5860	1.0263	−0.0278	0.115*	
C22	1.4367 (10)	0.8896 (6)	−0.00402 (19)	0.0584 (17)	
C23	1.5679 (9)	0.6105 (6)	0.0884 (2)	0.079 (2)	
H23A	1.6864	0.6008	0.0993	0.118*	
H23B	1.5732	0.6002	0.0605	0.118*	
H23C	1.4883	0.5510	0.0995	0.118*	
C24	1.4995 (9)	0.7333 (7)	0.0977 (2)	0.070 (2)	

### Atomic displacement parameters (Å^2^)

**Table:**

	*U*^11^	*U*^22^	*U*^33^	*U*^12^	*U*^13^	*U*^23^
N1	0.101 (5)	0.037 (3)	0.148 (5)	−0.011 (3)	0.056 (4)	−0.014 (3)
C1	0.098 (7)	0.224 (14)	0.104 (6)	0.023 (9)	−0.032 (6)	−0.029 (8)
O1	0.280 (13)	0.079 (4)	0.140 (5)	−0.068 (6)	0.113 (8)	−0.038 (4)
C2	0.100 (6)	0.062 (5)	0.079 (5)	0.001 (5)	0.003 (4)	0.005 (4)
N2	0.115 (8)	0.045 (4)	0.212 (10)	−0.009 (5)	0.088 (8)	−0.011 (6)
O2	0.076 (5)	0.080 (5)	0.379 (16)	−0.002 (4)	0.097 (8)	−0.002 (7)
N3	0.067 (4)	0.048 (3)	0.073 (3)	0.004 (3)	0.026 (3)	0.007 (3)
O3	0.088 (4)	0.063 (3)	0.147 (5)	−0.010 (3)	0.068 (4)	−0.007 (3)
C3	0.141 (9)	0.128 (9)	0.148 (9)	−0.052 (9)	−0.028 (8)	−0.006 (7)
N4	0.058 (3)	0.050 (3)	0.071 (3)	−0.014 (3)	−0.001 (3)	0.010 (3)
O4	0.123 (5)	0.045 (3)	0.145 (5)	−0.001 (3)	0.073 (4)	−0.011 (3)
C4	0.104 (7)	0.072 (5)	0.124 (7)	−0.013 (6)	−0.006 (6)	0.009 (5)
O5	0.245 (10)	0.046 (3)	0.122 (5)	0.025 (5)	−0.041 (6)	0.004 (3)
C5	0.077 (5)	0.039 (3)	0.086 (4)	−0.008 (3)	0.026 (4)	−0.004 (3)
O6	0.078 (3)	0.039 (2)	0.062 (2)	−0.002 (2)	0.000 (2)	0.0058 (19)
C6	0.073 (5)	0.032 (3)	0.088 (5)	−0.011 (3)	0.032 (4)	−0.010 (3)
C7	0.056 (4)	0.039 (4)	0.119 (6)	−0.012 (3)	0.025 (4)	0.002 (4)
O7	0.061 (3)	0.039 (2)	0.072 (3)	−0.003 (2)	0.001 (2)	−0.004 (2)
C8	0.058 (4)	0.035 (3)	0.070 (4)	−0.006 (3)	0.018 (4)	0.000 (3)
O8	0.096 (4)	0.066 (3)	0.101 (4)	−0.001 (3)	0.007 (3)	−0.026 (3)
O9	0.050 (2)	0.037 (2)	0.087 (3)	0.000 (2)	−0.007 (2)	−0.007 (2)
C9	0.057 (4)	0.028 (3)	0.064 (4)	−0.005 (3)	0.000 (3)	0.006 (3)
O10	0.097 (5)	0.076 (4)	0.210 (7)	−0.011 (4)	−0.072 (5)	−0.023 (5)
C10	0.048 (4)	0.039 (3)	0.077 (4)	−0.006 (3)	0.003 (3)	0.006 (3)
O11	0.070 (3)	0.045 (2)	0.073 (3)	0.009 (2)	0.006 (2)	0.003 (2)
C11	0.052 (4)	0.037 (3)	0.084 (4)	0.002 (3)	0.020 (3)	−0.001 (3)
C12	0.084 (5)	0.045 (4)	0.118 (6)	0.007 (4)	0.058 (5)	−0.007 (4)
C13	0.092 (5)	0.033 (3)	0.085 (5)	−0.009 (4)	0.027 (4)	0.006 (3)
C14	0.059 (4)	0.034 (3)	0.078 (4)	−0.002 (3)	0.008 (4)	−0.002 (3)
C15	0.065 (4)	0.051 (4)	0.078 (4)	0.004 (4)	0.016 (4)	0.003 (3)
C16	0.063 (4)	0.033 (3)	0.068 (4)	0.000 (3)	0.000 (3)	−0.002 (3)
C17	0.044 (3)	0.034 (3)	0.071 (4)	−0.007 (3)	−0.008 (3)	0.009 (3)
C18	0.051 (4)	0.028 (3)	0.089 (4)	0.000 (3)	−0.001 (4)	0.001 (3)
C19	0.122 (7)	0.058 (5)	0.135 (7)	−0.013 (5)	−0.033 (7)	0.030 (5)
C20	0.104 (7)	0.038 (4)	0.100 (6)	−0.006 (4)	−0.024 (5)	0.016 (4)
C21	0.084 (5)	0.063 (4)	0.082 (5)	−0.002 (4)	0.019 (4)	−0.005 (4)
C22	0.059 (4)	0.044 (4)	0.072 (4)	0.003 (3)	−0.001 (4)	−0.004 (3)
C23	0.046 (4)	0.069 (5)	0.121 (6)	0.006 (4)	−0.002 (4)	0.012 (4)
C24	0.044 (4)	0.059 (4)	0.107 (5)	−0.005 (4)	−0.014 (4)	0.014 (4)

### Geometric parameters (Å, °)

**Table:**

N1—C6	1.367 (8)	O8—C22	1.199 (8)
N1—C5	1.455 (9)	O9—C24	1.355 (8)
N1—H1A	0.8600	O9—C18	1.433 (7)
C1—C2	1.500 (10)	C9—C10	1.345 (8)
C1—H1B	0.9600	C9—C13	1.533 (8)
C1—H1C	0.9600	O10—C24	1.177 (8)
C1—H1D	0.9600	C10—C11	1.396 (8)
O1—N2	1.253 (15)	C10—H10A	0.9300
C2—C5	1.512 (10)	O11—C15	1.415 (7)
C2—H2A	0.9700	O11—C14	1.463 (7)
C2—H2B	0.9700	C12—H12A	0.9600
N2—O2	1.178 (15)	C12—H12B	0.9600
N2—C7	1.458 (10)	C12—H12C	0.9600
N3—O3	1.176 (7)	C13—H13A	0.9700
N3—O4	1.238 (7)	C13—H13B	0.9700
N3—C11	1.450 (8)	C14—C18	1.522 (9)
C3—C4	1.484 (13)	C14—H14A	0.9800
C3—H3A	0.9600	C15—C16	1.513 (8)
C3—H3B	0.9600	C15—H15A	0.9700
C3—H3C	0.9600	C15—H15B	0.9700
N4—C14	1.393 (8)	C16—C17	1.540 (8)
N4—C13	1.465 (9)	C16—H16A	0.9800
N4—H4A	0.8600	C17—C18	1.503 (8)
C4—C5	1.538 (11)	C17—H17A	0.9800
C4—H4B	0.9700	C18—H18A	0.9800
C4—H4C	0.9700	C19—C20	1.427 (11)
O5—C20	1.231 (9)	C19—H19A	0.9600
C5—H5A	0.9800	C19—H19B	0.9600
O6—C20	1.359 (8)	C19—H19C	0.9600
O6—C16	1.436 (7)	C21—C22	1.509 (10)
C6—C7	1.416 (9)	C21—H21A	0.9600
C6—C11	1.446 (9)	C21—H21B	0.9600
C7—C8	1.386 (8)	C21—H21C	0.9600
O7—C22	1.339 (7)	C23—C24	1.482 (10)
O7—C17	1.437 (7)	C23—H23A	0.9600
C8—C9	1.395 (8)	C23—H23B	0.9600
C8—C12	1.536 (9)	C23—H23C	0.9600
			
C6—N1—C5	132.8 (6)	C8—C12—H12C	109.5
C6—N1—H1A	113.6	H12A—C12—H12C	109.5
C5—N1—H1A	113.6	H12B—C12—H12C	109.5
C2—C1—H1B	109.5	N4—C13—C9	112.4 (6)
C2—C1—H1C	109.5	N4—C13—H13A	109.1
H1B—C1—H1C	109.5	C9—C13—H13A	109.1
C2—C1—H1D	109.5	N4—C13—H13B	109.1
H1B—C1—H1D	109.5	C9—C13—H13B	109.1
H1C—C1—H1D	109.5	H13A—C13—H13B	107.9
C1—C2—C5	115.8 (8)	N4—C14—O11	110.2 (5)
C1—C2—H2A	108.3	N4—C14—C18	112.1 (6)
C5—C2—H2A	108.3	O11—C14—C18	107.6 (4)
C1—C2—H2B	108.3	N4—C14—H14A	109.0
C5—C2—H2B	108.3	O11—C14—H14A	109.0
H2A—C2—H2B	107.4	C18—C14—H14A	109.0
O2—N2—O1	127.1 (11)	O11—C15—C16	109.3 (5)
O2—N2—C7	120.6 (13)	O11—C15—H15A	109.8
O1—N2—C7	112.2 (12)	C16—C15—H15A	109.8
O3—N3—O4	120.3 (6)	O11—C15—H15B	109.8
O3—N3—C11	119.1 (5)	C16—C15—H15B	109.8
O4—N3—C11	120.5 (6)	H15A—C15—H15B	108.3
C4—C3—H3A	109.5	O6—C16—C15	113.1 (5)
C4—C3—H3B	109.5	O6—C16—C17	105.8 (4)
H3A—C3—H3B	109.5	C15—C16—C17	108.4 (5)
C4—C3—H3C	109.5	O6—C16—H16A	109.8
H3A—C3—H3C	109.5	C15—C16—H16A	109.8
H3B—C3—H3C	109.5	C17—C16—H16A	109.8
C14—N4—C13	116.4 (6)	O7—C17—C18	108.1 (5)
C14—N4—H4A	121.8	O7—C17—C16	107.4 (4)
C13—N4—H4A	121.8	C18—C17—C16	110.1 (5)
C3—C4—C5	112.4 (7)	O7—C17—H17A	110.4
C3—C4—H4B	109.1	C18—C17—H17A	110.4
C5—C4—H4B	109.1	C16—C17—H17A	110.4
C3—C4—H4C	109.1	O9—C18—C17	107.1 (5)
C5—C4—H4C	109.1	O9—C18—C14	108.4 (4)
H4B—C4—H4C	107.9	C17—C18—C14	111.7 (5)
N1—C5—C2	107.8 (6)	O9—C18—H18A	109.8
N1—C5—C4	107.8 (6)	C17—C18—H18A	109.8
C2—C5—C4	112.1 (6)	C14—C18—H18A	109.8
N1—C5—H5A	109.7	C20—C19—H19A	109.5
C2—C5—H5A	109.7	C20—C19—H19B	109.5
C4—C5—H5A	109.7	H19A—C19—H19B	109.5
C20—O6—C16	118.1 (5)	C20—C19—H19C	109.5
N1—C6—C7	125.9 (6)	H19A—C19—H19C	109.5
N1—C6—C11	121.0 (6)	H19B—C19—H19C	109.5
C7—C6—C11	112.8 (5)	O5—C20—O6	117.9 (7)
C8—C7—C6	124.8 (6)	O5—C20—C19	128.1 (7)
C8—C7—N2	116.7 (6)	O6—C20—C19	113.7 (7)
C6—C7—N2	118.5 (6)	C22—C21—H21A	109.5
C22—O7—C17	118.2 (5)	C22—C21—H21B	109.5
C7—C8—C9	119.1 (6)	H21A—C21—H21B	109.5
C7—C8—C12	119.9 (6)	C22—C21—H21C	109.5
C9—C8—C12	121.0 (5)	H21A—C21—H21C	109.5
C24—O9—C18	119.5 (5)	H21B—C21—H21C	109.5
C10—C9—C8	119.3 (5)	O8—C22—O7	123.7 (7)
C10—C9—C13	121.2 (6)	O8—C22—C21	124.8 (7)
C8—C9—C13	119.5 (6)	O7—C22—C21	111.5 (6)
C9—C10—C11	122.3 (6)	C24—C23—H23A	109.5
C9—C10—H10A	118.8	C24—C23—H23B	109.5
C11—C10—H10A	118.8	H23A—C23—H23B	109.5
C15—O11—C14	111.7 (5)	C24—C23—H23C	109.5
C10—C11—C6	121.6 (6)	H23A—C23—H23C	109.5
C10—C11—N3	116.4 (6)	H23B—C23—H23C	109.5
C6—C11—N3	122.0 (5)	O10—C24—O9	121.1 (7)
C8—C12—H12A	109.5	O10—C24—C23	127.6 (7)
C8—C12—H12B	109.5	O9—C24—C23	111.2 (7)
H12A—C12—H12B	109.5		
			
C6—N1—C5—C2	−145.8 (9)	O4—N3—C11—C6	2.2 (10)
C6—N1—C5—C4	93.0 (11)	C14—N4—C13—C9	95.8 (7)
C1—C2—C5—N1	68.2 (9)	C10—C9—C13—N4	0.9 (9)
C1—C2—C5—C4	−173.4 (7)	C8—C9—C13—N4	−178.2 (5)
C3—C4—C5—N1	−170.6 (8)	C13—N4—C14—O11	66.0 (6)
C3—C4—C5—C2	71.0 (10)	C13—N4—C14—C18	−174.1 (5)
C5—N1—C6—C7	9.7 (15)	C15—O11—C14—N4	−174.5 (5)
C5—N1—C6—C11	−176.7 (7)	C15—O11—C14—C18	63.1 (6)
N1—C6—C7—C8	177.5 (8)	C14—O11—C15—C16	−66.7 (6)
C11—C6—C7—C8	3.4 (12)	C20—O6—C16—C15	91.4 (7)
N1—C6—C7—N2	−5.1 (14)	C20—O6—C16—C17	−150.0 (6)
C11—C6—C7—N2	−179.2 (9)	O11—C15—C16—O6	177.2 (4)
O2—N2—C7—C8	85.5 (12)	O11—C15—C16—C17	60.2 (6)
O1—N2—C7—C8	−91.0 (9)	C22—O7—C17—C18	114.5 (6)
O2—N2—C7—C6	−92.1 (11)	C22—O7—C17—C16	−126.7 (5)
O1—N2—C7—C6	91.4 (10)	O6—C16—C17—O7	66.9 (6)
C6—C7—C8—C9	−5.2 (12)	C15—C16—C17—O7	−171.5 (5)
N2—C7—C8—C9	177.4 (9)	O6—C16—C17—C18	−175.6 (5)
C6—C7—C8—C12	177.2 (8)	C15—C16—C17—C18	−54.1 (6)
N2—C7—C8—C12	−0.3 (12)	C24—O9—C18—C17	125.1 (6)
C7—C8—C9—C10	4.1 (10)	C24—O9—C18—C14	−114.2 (6)
C12—C8—C9—C10	−178.3 (6)	O7—C17—C18—O9	−70.9 (6)
C7—C8—C9—C13	−176.8 (7)	C16—C17—C18—O9	172.1 (4)
C12—C8—C9—C13	0.8 (10)	O7—C17—C18—C14	170.5 (5)
C8—C9—C10—C11	−1.7 (10)	C16—C17—C18—C14	53.5 (6)
C13—C9—C10—C11	179.2 (7)	N4—C14—C18—O9	64.8 (7)
C9—C10—C11—C6	0.2 (10)	O11—C14—C18—O9	−173.9 (5)
C9—C10—C11—N3	−179.7 (6)	N4—C14—C18—C17	−177.4 (5)
N1—C6—C11—C10	−175.3 (7)	O11—C14—C18—C17	−56.1 (6)
C7—C6—C11—C10	−0.9 (10)	C16—O6—C20—O5	−12.9 (12)
N1—C6—C11—N3	4.6 (11)	C16—O6—C20—C19	172.6 (7)
C7—C6—C11—N3	179.0 (7)	C17—O7—C22—O8	−4.5 (9)
O3—N3—C11—C10	0.2 (10)	C17—O7—C22—C21	175.2 (5)
O4—N3—C11—C10	−177.9 (7)	C18—O9—C24—O10	−1.5 (10)
O3—N3—C11—C6	−179.7 (7)	C18—O9—C24—C23	−178.9 (6)

### Hydrogen-bond geometry (Å, °)

**Table:**

*D*—H···*A*	*D*—H	H···*A*	*D*···*A*	*D*—H···*A*
N1—H1A···O4	0.86	1.92	2.621 (8)	138
